# Impact of case management by advanced practice nurses in primary care on unplanned hospital admissions: a controlled intervention study

**DOI:** 10.1186/1472-6963-8-115

**Published:** 2008-05-29

**Authors:** Dyfed W Huws, Deborah Cashmore, Robert G Newcombe, Catherine Roberts, Judith Vincent, Glyn Elwyn

**Affiliations:** 1 Department of Primary Care and Public Health, School of Medicine, Cardiff University, Neuadd Meirionnydd, Heath Park, Cardiff, CF14 4YS, UK; 2 Swansea Local Health Board, Kidwelly House, Charter Court, Phoenix Way, Swansea, SA7 9FS, Wales, UK

## Abstract

**Background:**

Increasing unplanned hospital admissions disrupt planned health care, lead to additional morbidity and are expensive. A recent review found only weak evidence for case management preventing unplanned admissions, yet case management of older people is being implemented widely in the UK. We aimed to study the effect of advanced practice nurse case management on unplanned medical and geriatric hospital admission rates in patients 50 years and over, and on admission risk in a 'higher risk' sub-group of patients in the UK.

**Methods:**

Case management by advanced practice nurses in NHS primary care practices in the Swansea Local Health Board area, Wales, UK. We conducted a prospective non-randomized controlled intervention study comparing unplanned medical and geriatric patient admissions between five intervention and thirty non-intervention practices during a pre-intervention year and an intervention year.

**Results:**

For all lengths of stay, comparing intervention (n = 5) with non-intervention practices (n = 30) from pre-intervention to intervention year, we found that the unplanned medical and geriatric admission rate was significantly lower in the intervention group – adjusted relative risk of 0.909; relative risk reduction 9.1% (95% credible limit 0.840 to 0.984, p = 0.018); absolute risk reduction 0.99 admissions per 100 patients (95% credible limit 0.17 to 1.86, p = 0.018). For lengths of stay of one night or more we observed a stronger effect – adjusted relative risk 0.896; relative risk reduction 10.41% (95%, credible limit 0.820 to 0.979, p = 0.015). Most of the rate reduction was due to a reduction in the number of new admissions but much less so for admissions of lengths of stay of at least one night, compared to all lengths of stay. We did not find a statistically significant effect on re-admission or multiple re-admission rates in 'higher risk' patients previously admitted one or more times – adjusted relative risk of further multiple admissions per previously admitted patient 0.908 (95% credible limit 0.765, 1.077); relative risk reduction 9.3%; adjusted relative risk of total admissions per multiple admitter 0.995 (95% credible limit 0.940, 1.053) relative risk reduction 0.6%.

**Conclusion:**

Although this study reports a reduction in unplanned admission rates in the intervention practices, this appears to be only in part directly due to nurse case management: most of the reduction did not occur in multipe admitters whom were case managed. Further research is needed to explain this finding, to elucidate how best to target the attention of case managers and to examine the complexity of potential outcomes in terms of the nature and necessity of admissions and most suitable lengths-of-stay in terms of acute care or rehabilittion need.

## Background

Interest in reducing the increasing numbers of unplanned hospital admissions in the UK, as in other countries [1-5], is leading to attempts to stem the demand [6]. Rising unplanned admissions are expensive and disrupt planned health care, notably by impeding elective work. They also cause considerable disruption for patients and carers. One of the approaches attempted has been case management. Case management was developed in the US and attempts to identify individuals judged to have an increased risk of unplanned hospital admission. Those identified are managed proactively, primarily in an attempt to reduce unplanned hospital admissions. However, few studies describe the intervention and the processes undertaken in detail [7,8].

Case management of frail older people by practice-based nurses with extended roles has been implemented widely in England through many initiatives and by the policy-based recruitment of 'community matrons' [9]. This policy was partly based on an American study which reported that case management of frail older people in long stay nursing homes in an Evercare programme appeared to halve the hospital admission rate over a 15 month period [8], and partly based on non-peer reviewed data from an Evercare pilot in England. However, an evaluation of the Evercare pilot of nurse case management with an extended generalised role, in 62 primary care practices across England, did not identify a significant effect on unplanned admission, length of patient stay in hospital or mortality [10]. A recent review of case management studies found only weak evidence for case management in preventing unplanned admissions and inconsistent effects on emergency department use [7]. Studies have been heterogeneous, often small and of variable quality. It may also be difficult to replicate some US interventions in the UK owing to different health and social care systems [11]. Despite uncertainty about the effect on hospital admissions, some evidence suggests that patient and carer satisfaction and quality of life may be improved [12,13].

Despite the uncertainties, several primary care organisations – Primary Care Trusts (PCTs) in England and Local Health Boards (LHBs) in Wales – have implemented a case management approach. In Wales, unplanned admission rates are about 40% higher than in England [14], rising from being accountable for 51% of all hospital admissions in 1997–8 to 58% of all admissions in 2002–3 [15]. The Welsh Assembly Government responded by setting a NHS target for 2006–7 of a 10% reduction in reported emergency medical re-admissions, compared to 2004–5 [16]. LHBs responded by searching for interventions that they hoped would address this issue and we report a study commissioned by the Swansea LHB. We report the findings of a study that aimed to examine the effect of advanced practice nurse (APN) case management on unplanned medical and geriatric hospital admission rates and lengths of hospital stay in patients 50 years and older, and in particular the impact on a sub-group of patients identified as being at 'higher risk' of unplanned admission.

## Methods

### Study design

We conducted a prospective intervention study by comparing unplanned medical and geriatric patient admissions between intervention and non-intervention practices during a pre-intervention year (1 April 2004 to 31 March 2005) and an intervention year (1 April 2005 to 31 March 2006). We used the patient population that was aged 50 years and over in all practices and compared admissions and lengths of stay in hospital across two dimensions: comparing admission rates in the same practice groups between the two years (pre-intervention against invention year) and secondly admission rates between practice groups at the same time point – in essence, within-group and across-group comparisons. We also studied the intervention's effect on annual re-admission risk in an apparently higher risk sub-group of people who had already undergone multiple admissions in the preceding year. We excluded admissions to community hospitals, elective admissions, and all other admission types. The study received ethics committee approval from the Swansea Local Research Ethics Committee and written informed consent was obtained from participants.

### The intervention

APNs were recruited to the newly created role and employed by the LHB. APNs were provided with an 8-week induction course that included history-taking, diagnostic skills, visits to patients, practices, voluntary agencies, social services, and intermediate care and rehabilitation units. APNs were led by a senior nurse-manager employed by the LHB. APNs were each allocated to one practice. Following a process of selecting patients (see below), individually tailored case management packages were agreed with the patients and their carers. Packages could include self-help advice, carer support, coordination of inputs from voluntary and statutory organisations, and planned primary and secondary health care. In theory, such interventions and services could be available to other non-intervention practices, but APNs at the intervention practice could intensely assess need and proactively coordinate such inputs. APNs adapted the content, intensity and duration of packages of care as patients' needs changed.

### Practice and patient selection

The LHB invited nine Swansea practices from an existing collaborative consortium to consider receiving case-management APNs and five accepted. Patients aged 50 years and over were the designated denominator population. Similar patients registered with all the remaining practices in Swansea formed the control population in what we call here non-intervention practices.

The criteria for initial screening of patients by the APNs during the intervention year were either a history of 2 or more admissions in the pre-intervention year and/or a new unplanned admission during the intervention year. At the start of the study APNs were given the names of patients in their respective practices who had two or more admissions in the pre-intervention year identified by Patient Episode Database Wales (PEDW) hospital activity data. During the intervention year we also informed APNs each week about patients admitted in the preceding week using data from Swansea NHS Trust. No such information was provided for non-intervention practices.

Patients notified to APNs were screened for case management suitability. Screening consisted of a clinical interview, medication review, consideration of social circumstances and functioning, and judging the risk of re-admission. This process was typically achieved by a home visit or telephone call. This was done early in the study for patients identified in the preceding year, and immediately post-discharge for patients identified during the intervention year. APNs could liaise with a nominated general practitioner if necessary. Once screened and accepted for case management, APNs allocated patients to high to medium to low unplanned re-admission risk categories, and then moved patients between different categories and so varied their inputs accordingly. APNs also accepted referrals of other patients considered to be at potentially increased risk from GPs and other healthcare professionals.

### Quantitative analysis

The analysis of admission data is complex because patients often have multiple admissions in any given timeframe: the nested nature of the data needs meticulous consideration. For this reason, we have developed a notation for numerators and denominators – see Table 1 for details. We calculated the denominator population aged 50 years and over (n), for each intervention and non-intervention practice, for pre-intervention and intervention years, using monthly practice registration data. By reconciling Swansea NHS Trust admission data with PEDW data, we determined (e) and (r) for intervention and non-intervention practices.

**Table 1:** Notation used for calculated parameters and outcome measures

n = denominator population patients aged 50 years and over
a = number of unplanned hospital medical or geriatric admissions
e = number of people ever admitted
r = number of people who admitted more than once

a/n = unplanned hospital admission per denominator population
e/n = proportion of denominator population ever admitted
(e-r)/n = single admitters as a proportion of the denominator population
(r/n) = multiple admitters as a proportion of the denominator population
(r/e) = multiple admissions per admitted patient
*(a-e)/e *= re-admissions per admitted patient
(a-e)/a = re-admissions as a proportion of all admissions
(a-e+r)/r = total admissions per multiple admitter
(a-e)/r = re-admissions per multiple admitter
(a-e-r)/r = 3rd and higher order admissions per multiple admitter

For admissions of all lengths of stay, we calculated the rate in the denominator population (a/n) both in intervention and non-intervention practices, for pre-intervention and intervention years. We estimated the adjusted relative risk by dividing the relative change in rate from pre-intervention to intervention year for intervention practices by that for non-intervention practices (a 'ratio of ratios' approach). We used an additive model to calculate absolute risk difference for the rate change – a 'difference of differences' approach. These were the two main outcome measures.

We did not match or adjust for differences between intervention and non-intervention practices as we allowed for all unobserved differences between practices, assuming no change between pre-intervention and intervention years. We did not use mortality data. We fitted Bayesian regression models using WinBugs software [17], based on a Poisson distribution, and allowed for practice-level variation (over-dispersion) by adding a random-effects term to the transformed rate, following log transformation. Adjusted relative risks were thus calculated, with tail-based 95% credible intervals, and 2-sided p-values derived by doubling the posterior probability of obtaining a ratio of 1 or above.

Similarly, we calculated the ratio of ratios for the number of people ever admitted in the denominator population (e/n), but based on a binomial distribution. Using similar analyses, we split this outcome into of people who had been admitted on one occasion only (e-r)/n and those who had multiple admissions as a proportion of the denominator population (r/n).

We also examined the same year re-admission risk in those who had been identified as having had an existing admission. We calculated the ratio of ratios of multiple admissions per admitted patient (r/e), for intervention and non-intervention practices, both for pre-intervention and intervention years. We repeated this process for re-admissions per admitted patient (a-e)/e; re-admissions as a proportion of all admissions (a-e)/a; total admissions per multiple admitter (a-e+r)/r; re-admissions per multiple admitter (a-e)/r; and 3rd and higher order admissions per multiple admitter (a-e-r)/r. The main outcome measure – the annual admission rate (a/n) – has three components: (e/n); (r/e); and (a-e+r)/r. We examined the relative contribution of these components to the observed changes in a/n.

We repeated these analyses for admissions with a length of stay of at least one night or more, excluding assessments (also counted as 'admissions') sent home the same day. Separately, we also compared the mean length of stay for pre-intervention and intervention years for intervention and non-intervention practices, assigning 'admissions' with no overnight stay a value of 0.5 days.

## Results

Five practices accepted the invitation to take part in the intervention arm. The remaining 30 practices in Swansea formed the non-intervention practices. Five APNs were appointed (4.5 full time equivalents), two from primary care nursing backgrounds and three from hospital settings. APNs were allocated to specified practices, sometimes providing cross-cover for annual leave. All APNs underwent an induction programme as planned. During the intervention year the APNs met on a weekly basis.

In the pre-intervention year, 1496 admissions occurred from the intervention practices: 1095 patients from the intervention practices' denominator population. Of these, 255 patients were re-admitted and so had two or more admissions in the pre-intervention year. APNs were notified of these patients at the beginning of the intervention year. During the intervention year, 1401 admissions occurred: 1034 patients were admitted from the intervention practices' denominator population. Of these 226 patients were re-admitted in the same intervention year. During the intervention year, APNs were notified about all 1401 admissions and re-admissions the week after they occurred.

The main quantitative results for all lengths of stay are shown in Table 2. From pre-intervention to intervention year, the main outcome measure (a/n) decreased by 6.8% from 0.1104 to 0.1029 in intervention practices, but increased by 2.5% from 0.1107 to 0.1135 in non-intervention practices, giving an adjusted relative risk (RR) of 0.909 (95% credible limit 0.840 to 0.984, p = 0.018), and a RR reduction of 9.1%. The corresponding annual absolute risk reduction (ARR) was 0.99 admissions per 100 patients (95% credible limit 0.17 to 1.86, p = 0.018). For the 13619 intervention practices' denominator population we estimate this to be a reduction of 135 admissions per year, equivalent to 30 less admissions per APN full time equivalent.

**Table 2:** Adjusted 'ratio of ratios' for all outcome measures, comparing practices across groups and between pre-intervention and intervention years

							**With adjustment for overdispersion**			
							
**Group**	**Quantity**	**Intervention practices**		**Non-intervention practices**		**Crude relative risk**	**Point estimate**	**95% credible limits**		**P-value**
						
		**2004–5**	**2005–6**	**2004–5**	**2005–6**			**Lower**	**Upper**	
**Population (50 years and older)**	n	13556	13619	72492	72675					
**Admissions**	a	1496	1401	8025	8246					
**Ever admitted**	e	1095	1034	5954	5998					
**Re-admitted**	r	255	226	1365	1421					
**Analysis**										
Admissions/registered patient	a/n	0.1104	0.1029	0.1107	0.1135	**0.909**	**0.909**	**0.841**	**0.984**	**0.018**
Proportion registered patients ever admitted in year	e/n	0.0808	0.0759	0.0821	0.0825	**0.935**	**0.935**	**0.855**	**1.022**	**0.138**
Single admitters as proportion of all registered patients	(e-r)/n	0.0620	0.0593	0.0633	0.0630	**0.962**	**0.962**	**0.869**	**1.065**	**0.457**
Re-admitters as a proportion of all registered patients	r/n	0.0188	0.0166	0.0188	0.0196	**0.850**	**0.850**	**0.700**	**1.030**	**0.097**
Proportion admitted patients with multiple admissions	r/e	0.233	0.219	0.229	0.237	**0.908**	**0.908**	**0.765**	**1.078**	**0.265**
Total admissions per re-admitter	(a-e+r)/r	2.573	2.624	2.517	2.582	**0.994**	**0.995**	**0.940**	**1.053**	**0.820**
Re-admissions per re-admitter	(a-e)/r	1.573	1.624	1.517	1.582	**0.990**	**0.991**	**0.903**	**1.088**	
3^rd ^& higher order admissions per re-admitter	(a-e-r)/r	0.573	0.624	0.517	0.582	**0.968**	**0.971**	**0.753**	**1.251**	
Re-admissions per admitter	(a-e)/e	0.366	0.355	0.348	0.375	**0.900**	**0.900**	**0.772**	**1.050**	**0.181**
Re-admissions as a proportion of all admissions	(a-e)/a	0.268	0.262	0.258	0.273	**0.925**	**0.926**	**0.826**	**1.037**	

From pre-intervention to intervention year, (e/n) reduced by 6.0% from 0.0808 to 0.0759 in intervention practices. In contrast, e/n increased by 0.5% from 0.0821 to 0.0825 in non-intervention practices, leading to an adjusted RR of 0.935 (95% credible limit 0.855, 1.02, p = 0.14), and RRR of 6.5%.

For patients identified as having an admission(s) in the preceding year, multiple admissions per admitted patient (r/e) reduced by 6.1% from the pre-intervention to the intervention year in the intervention practices, compared to a 3.3% increase in non-intervention practices, giving an adjusted RR of 0.908 (95% CI 0.765, 1.077), and a RRR of 9.3%. The (a-e+r)/r increased by 2.0% from pre-intervention to intervention year in intervention practices, and by 2.6% in non-intervention practices, leading to an adjusted RR of 0.995 (95% CI 0.940, 1.053), and a RRR of 0.6%. None of these differences were statistically significant at the 5% level.

Further analyses indicated that about 70% of the RRR in a/n for all lengths of stay was attributable to a reduction in e/n. 30% of the RRR in a/n was attributable to the effect on re-admissions among those admitted once or more than once, made up of 27% attributed to a reduction in r/e, and 2% attributed to (a-e+r)/r.

In the intervention practices, the mean length of stay following unplanned admissions reduced from 1.098 days per patient in the pre-intervention year to 0.911 days per registered patient in intervention, a relative reduction of 17%. There was a less marked reduction in non-intervention practices, from 1.0525 days per patient to 0.9917 days per registered patient, a relative reduction of 6.0%. The RR was 0.881, corresponding to a RRR of 11.9%.

### Length of stay of one night or more

For the main outcome of a/n, the adjusted RR comparing intervention and non-intervention practices from pre-intervention to intervention years was 0.896 (95% credible limit 0.820 to 0.979, p = 0.015), giving a RRR of 10.41% – where 67% of the improvement in a/n is attributable to a reduction in e/n, 24% to a reduction in r/e, and 10% to a reduction in (a-e+r)/r.

## Discussion

### Principal findings

This controlled study of nurse case management implemented in a real world primary care context demonstrated a statistically significant relative risk reduction of 9.1% in unplanned medical and geriatric admission rate in patients aged 50 years. This reduction was observed in intervention practices compared to non-intervention practices and from pre-intervention to intervention years. For the intervention practices' denominator population, we estimate this to be a reduction of 135 admissions per year, equivalent to 30 less admissions per full time equivalent APN. A reduction in the proportion of admitted patients going on to re-admit in the same year was also observed but this was not statistically significant. Most of the reduction in the main outcome measure was due to an effect on new admissions: less than a third of the reduction was attributable to the effect on re-admissions among those admitted once or more than once. Only 2% was attributable to a reduction in further same year re-admissions amongst multiple admitters. For admissions that included at least one night's hospital stay, the relative risk reduction in the main outcome measure was slightly larger than for admissions of all lengths of stay. The contribution of same year re-admissions amongst multiple admitters to the reduction in the main outcome measure was much greater than for all lengths of stay.

### Strengths and weaknesses of the study

There may well be alternative explanations for the observed RRR in intervention practices apart from APN case management. This is very likely as most of the reduction in the main outcome measure was due to an effect on new admissions rather than on re-admissions among those admitted once or more than once. In general, APNs did not case manage those without a recent admission or multiple admissions.

The study has several limitations. Given service development constraints, we were not able to set a sampling frame or to randomise practices to the intervention. The small number of APNs and intervention practices limited the power of the study, even by using control data from all other 30 LHB practices and we would have preferred to analyse data for a longer duration. The placement of the APNs in a selected group of practices may well have resulted in bias and contributed to the observed effect in the intervention practices. For example, it may be possible that the presence of the APN s made other clinical staff at the intervention practices more aware of the risk of admission in their older patients without a history of admission. They may have then carried out interventions similar to case management. However this was not assessed in out study. Without randomisation, we were unable to control for other factors, such as improvements in chronic condition management that might have existed in these organisations. We did not analyse mortality data, and it is possible that a difference in mortality between the two groups could have affected our calculation of admission rates.

Our study however had many strengths. The study was pragmatic – it had the benefit of being exactly what was feasible in a health service under constraints. The intervention we used could be easily repeated within the existing NHS system elsewhere. We ensured that we had two levels of control by simultaneously comparing intervention with non-intervention practices, and from pre-intervention to intervention year. We allowed for the effect of practice level intervention and not the individual level in our analysis, using Bayesian techniques where appropriate. In addition we considered both the overall registered population and those already previously admitted as appropriate denominators for our outcome measures.

### Results in context

Unlike our study, the recent larger evaluation of an Evercare case management approach in England showed no statistically significant reduction in unplanned admission rate or length of stay, both overall and amongst multiple admitters [10]. In fact, the trend was towards increased admissions and longer length of stay among 'high risk' multiple admitters in intervention practices. APNs might discover through case finding, unmet need and thus increase unplanned admissions, at least in the short-term. Likewise, APN intervention could result in additional unplanned admissions – which would otherwise be deaths – or result in elective admissions sooner, rather than unplanned ones later. We know that a high mortality amongst one large cohort of multiple admitters [18] could have explained the observed reduction in re-admission rate with time in the absence of any intervention.

Other differences in study design may also explain differences in our results compared to the Evercare study [10]. Chance is a possible explanation, given our smaller scale study. Another is that APNs could intervene earlier in our study because they also received contemporaneous data about recent admissions, as well as information on those admitted twice or more in the preceding year. It is also possible that the younger age group in our study may have responded better to case management. We analysed the effect on medical or geriatric admissions whereas all unplanned admissions were included in the English study. Finally, the underlying unplanned admission rate is higher in Wales than in England [19], so it is possible the intervention may have had more scope to show an effect.

Despite our acknowledged study weaknesses, which are similar to weaknesses in other studies [7,10,11], we have demonstrated that APN case management resulted in a reduction in unplanned admissions. Uncertain results and diminished effectiveness in published studies may arise from a problem in the identification of patients at risk of admission – case risk attribution. In this and in other studies [20], case risk attribution tools have relied heavily on past admission as the main risk factor for identifying patients where case management was thought to have the main potential benefit, even though this now appears to be a poor predictor of future admissions in the long term, and that most unplanned admissions arise in those not recently admitted [18]. We are also aware of other positive results where case management resulted in a statistically significant reduction in hospitalisation amongst frail older people in the community, for example in a randomized study in northern Italy [21].

## Conclusion

Although this study reports a reduction in unplanned admission rates in the intervention practices this appears to be only in part directly due to nurse case management. There needs to be further work to confirm and explore further our findings and to consider the cost-effectiveness of APN case management. We do not know the range of risk factors that influence unplanned admission rates and we are as yet unable to predict for which patients exactly interventions are best targeted in order to prevent 'avoidable' admissions. Uncertainty persists, particularly about the utility of case management that does not provide the intensive 'hospital at home' facilities which are part of the Evercare and similar health maintenance organisation-based approaches. However, although caution needs to be advocated, it is important not to dismiss case management – there is emerging evidence that the approach addresses a significant and unattended care gap and, when well-implemented, appears to improve the patient's quality of life [12].

## Competing interests

The study and evaluation was funded by the Swansea Local Health Board. CR and JV are employees of Swansea Local Health Board and DC was funded by them. The authors declare that they have no competing interests.

## Authors' contributions

All authors contributed to the design of the study, commented on the analysis, contributed to writing the paper, read and approved the final manuscript. GE is the guarantor of the paper.

## Pre-publication history

The pre-publication history for this paper can be accessed here:


